# Variants in the DC-SIGN–Encoding Gene *CD209* and Susceptibility to Tuberculosis

**DOI:** 10.1371/journal.pmed.0030061

**Published:** 2006-01-03

One-third of the world's population is thought to be infected with Mycobacterium tuberculosis, the bacterium that causes tuberculosis. Yet, even though 2–3 million people die from tuberculosis every year, only 5%–10% of individuals infected with the causative agent ever become ill. Many factors affect how well a person's immune system fights off an attack by M. tuberculosis, including HIV infection, age, and malnutrition. It is also becoming clear that, as with other infectious diseases, host genetic factors affect susceptibility to tuberculosis.

One of the first genes to be associated with susceptibility to tuberculosis was *NRAMP1* (natural resistance-associated macrophage protein 1, now renamed *SLC11A1*). Others include the genes for the vitamin D receptor and the mannose receptor, and major histocompatibility complex (MHC) class II alleles. Variants of these genes, all of which encode proteins involved in the immune response, may affect how well the immune system deals with M. tuberculosis.

Luis Barreiro, Lluis Quintana-Murci, and colleagues now report that two variants in the gene encoding another immune system molecule—DC-SIGN, which stands for dendritic cell–specific ICAM-3-grabbing nonintegrin—are linked to tuberculosis susceptibility. The first rapid line of defense against M. tuberculosis and other pathogens is the innate immune system, where germline-encoded receptors recognize general features on pathogens. DC-SIGN, a C-type lectin that recognizes specific carbohydrate side chains present on the surface of pathogens, is the major receptor of this type for M. tuberculosis on human dendritic cells (immune system cells that process pathogens for presentation to the acquired immune system). Consequently, *CD209*—the gene encoding DC-SIGN—is a good place to search for genetic variants associated with tuberculosis susceptibility. [Fig pmed-0030061-g001]

**Figure pmed-0030061-g001:**
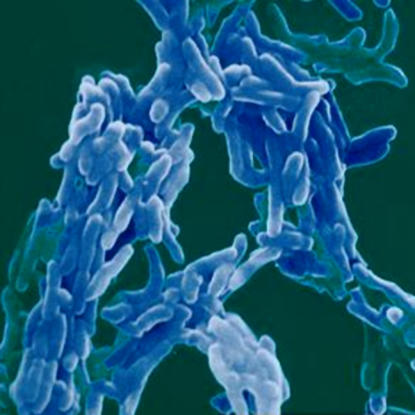
False-color image of Mycobacterium tuberculosis

The researchers looked for *CD209* gene variants in 351 individuals with tuberculosis and in 360 healthy controls living in the Cape Town area of South Africa. People living there are known as South African Colored and have a present-day uniform ethnicity. However, they derive genetically from populations of different ethnic backgrounds with different susceptibilities to tuberculosis. Due to the very high local incidence of tuberculosis, everyone living in this region, even healthy controls, is likely to have been exposed to M. tuberculosis. None of the participants were HIV-positive.

Barreiro and colleagues discovered two single-nucleotide polymorphisms (SNPs) or variants in *CD209* whose frequency differed significantly between healthy controls and people with tuberculosis. Both were in the promoter of *CD209* (promoters are noncoding regions that enable genes to be transcribed and proteins to be made). Variants –871G (a guanine 871 nucleotides upstream from the *CD209* coding region) and –336A (an adenosine 336 nucleotides upstream from the coding region), either alone or in combination, were associated with decreased risk of developing tuberculosis in the study population. To make sure that these associations reflected differences in disease susceptibility and not ethnic differences between the control and the diseased groups (a problem called population stratification), the researchers showed that 25 other SNPs located genomewide had similar frequencies in both groups.

The researchers also report that the “protective” variants of *CD209* are more prevalent in Eurasians than in Africans—their presence in the South African Colored population likely reflects the mixing of the two ethnic populations. They suggest that –871G and –336A may have been selected for in Europeans but not in Africans because tuberculosis has been endemic in Europe for several centuries, whereas in Africa it is thought to have arrived much more recently. However, because DC-SIGN is a receptor for many other pathogens, this geographical pattern of variants may reflect exposure to other infectious agents besides M. tuberculosis. Indeed, genetic variation at position –336 of the *CD209* promoter is associated with both the susceptibility to HIV and the severity of dengue fever. Whether it is the polymorphisms at positions –871 and –336 that directly affect host susceptibility (as opposed to other genetic variants of *CD209* that these polymorphisms are closely linked to) is not entirely clear; nor is it known how the variants affect host susceptibility to infectious disease. One possibility is that they alter how much DC-SIGN is made (and results by others suggest that this is the case for at least one of the variants), which in turn could change the effectiveness of the host immune response. Knowing the functional consequences of the *CD209* variants and the mechanisms by which they affect disease susceptibility, suggest the authors, should ultimately help in the development of new knowledge-based treatments for tuberculosis and other infectious diseases.

